# Human Leukocyte Antigens and Systemic Lupus Erythematosus: A Protective Role for the HLA-DR6 Alleles DRB1*13:02 and *14:03

**DOI:** 10.1371/journal.pone.0087792

**Published:** 2014-02-03

**Authors:** Hiroshi Furukawa, Aya Kawasaki, Shomi Oka, Ikue Ito, Kota Shimada, Shoji Sugii, Atsushi Hashimoto, Akiko Komiya, Naoshi Fukui, Yuya Kondo, Satoshi Ito, Taichi Hayashi, Isao Matsumoto, Makio Kusaoi, Hirofumi Amano, Tatsuo Nagai, Shunsei Hirohata, Keigo Setoguchi, Hajime Kono, Akira Okamoto, Noriyuki Chiba, Eiichi Suematsu, Masao Katayama, Kiyoshi Migita, Akiko Suda, Shigeru Ohno, Hiroshi Hashimoto, Yoshinari Takasaki, Takayuki Sumida, Shouhei Nagaoka, Naoyuki Tsuchiya, Shigeto Tohma

**Affiliations:** 1 Clinical Research Center for Allergy and Rheumatology, Sagamihara Hospital, National Hospital Organization, Sagamihara, Japan; 2 Molecular and Genetic Epidemiology Laboratory, Faculty of Medicine, University of Tsukuba, Tsukuba, Japan; 3 Department of Rheumatology, Tokyo Metropolitan Tama Medical Center, Fuchu, Japan; 4 Department of Rheumatology, Sagamihara Hospital, National Hospital Organization, Sagamihara, Japan; 5 Department of Internal Medicine, Faculty of Medicine, University of Tsukuba, Tsukuba, Japan; 6 Department of Rheumatology, Niigata Rheumatic Center, Shibata, Japan; 7 Department of Internal Medicine and Rheumatology, Juntendo University School of Medicine, Tokyo, Japan; 8 Department of Rheumatology and Infectious Disease, Kitasato University School of Medicine, Sagamihara, Japan; 9 Department of Allergy and Immunological Diseases, Tokyo Metropolitan Cancer and Infectious Diseases Center Komagome Hospital, Tokyo, Japan; 10 Department of Internal Medicine, Teikyo University, Tokyo, Japan; 11 Department of Rheumatology, Himeji Medical Center, National Hospital Organization, Himeji, Japan; 12 Department of Rheumatology, Morioka Hospital, National Hospital Organization, Morioka, Japan; 13 Department of Internal Medicine and Rheumatology, Clinical Research Institute, Kyushu Medical Center, National Hospital Organization, Fukuoka, Japan; 14 Department of Internal Medicine, Nagoya Medical Center, National Hospital Organization, Nagoya, Japan; 15 Clinical Research Center, Nagasaki Medical Center, National Hospital Organization, Omura, Japan; 16 Center for Rheumatic Diseases, Yokohama City University Medical Center, Yokohama, Japan; 17 Department of Rheumatology, Yokohama Minami Kyosai Hospital, Yokohama, Japan; 18 Juntendo University School of Medicine, Tokyo, Japan; Keio University School of Medicine, Japan

## Abstract

Many studies on associations between human leukocyte antigen (HLA) allele frequencies and susceptibility to systemic lupus erythematosus (SLE) have been performed. However, few protective associations with *HLA-DRB1* alleles have been reported. Here, we sought protective, as well as predispositional, alleles of *HLA-DRB1* in Japanese SLE patients. An association study was conducted for *HLA-DRB1* in Japanese SLE patients. Relative predispositional effects were analyzed by sequential elimination of carriers of each allele with the strongest association. We also explored the association of *DRB1* alleles with SLE phenotypes including the presence of autoantibody and clinical manifestations. Significantly different carrier frequencies of certain *DRB1* alleles were found to be associated with SLE as follows: increased DRB1*15:01 (*P* = 5.48×10^−10^, corrected *P* (*P*c) = 1.59×10^−8^, odds ratio [OR] 2.17, 95% confidence interval [CI] 1.69–2.79), decreased DRB1*13:02 (*P* = 7.17×10^−5^, *P*c = 0.0020, OR 0.46, 95% CI 0.34–0.63) and decreased DRB1*14:03 (*P* = 0.0010, *P*c = 0.0272, OR 0.34, 95% CI 0.18–0.63). Additionally, the “*15:01/*13:02 or *14:03” genotype tended to be negatively associated with SLE (*P* = 0.4209, OR 0.66), despite there being significant positive associations with *15:01 when present together with alleles other than *13:02 or *14:03 (*P* = 1.79×10^−11^, OR 2.39, 95% CI 1.84–3.10). This protective effect of *13:02 and *14:03 was also confirmed in SLE patients with different clinical phenotypes. To the best of our knowledge, this is the first report of a protective association between the carrier frequencies of HLA-DRB1*13:02 and *14:03 and SLE in the Japanese population.

## Introduction

Systemic lupus erythematosus (SLE) is a prototypic autoimmune disease of unknown etiology that affects multiple organs and is associated with the production of several different autoantibodies. It is a systemic inflammatory disease susceptibility to which is associated with genetic and environmental factors [1]. Genetic risk factors for SLE include alleles in *IRF5, STAT4, BLK, TNFAIP3, TNIP1, FCGR2B* and others [2], [3], [4]; the functional role of the polymorphisms as well as the relationships with other autoimmune diseases such as rheumatoid arthritis were suggested[5], [6]. Especially, altered frequencies of human leukocyte antigen (*HLA*) alleles are known to be associated with SLE. Some *HLA-DRB1* alleles are reported to be positively associated with SLE susceptibility in several ethnic groups studied: DRB1*03:01 and *15:01 in European [7], [8], *15:03 in African-American [9], *08:02 in Hispanic [10] and *15:01 and *15:02 in Asian populations [11], [12], [13], [14]. Gene dosage effects were not noted in the associations of *HLA-DRB1* alleles with susceptibility to SLE in that homozygosity for a susceptibility allele does not confer higher disease risk than heterozygosity for that allele. However, only limited information is available concerning protective *DRB1* alleles for SLE, i.e. those with a reduced frequency in patients. Here, we sought protective, as well as predispositional, *HLA-DRB1* alleles in Japanese SLE patients. We also explored associations of *DRB1* alleles with SLE phenotypes including the presence of autoantibody and clinical manifestations of disease.

## Materials and Methods

### Patients and controls

A first set of 459 SLE patients (with a mean age ± standard deviation (SD) of 48.2±15.4 years) of whom 36 were men was recruited at Sagamihara Hospital, Yokohama Minami Kyosai Hospital, Tama Medical Center, Kitasato University, Komagome Hospital, Teikyo University, Himeji Medical Center, Morioka Hospital, Kyushu Medical Center and Yokohama City University Medical Center and a second set of 389 patients (41.6±13.7 years of age; 31 men) at University of Tsukuba, Juntendo University and the University of Tokyo. A first set of 307 healthy controls (39.5±11.1 years; 2 men) was recruited at Sagamihara Hospital or by the Pharma SNP Consortium (Tokyo, Japan) [15] and a second set of 542 healthy controls (34.0±9.8 years; 245 men) at the University of Tokyo and University of Tsukuba. All patients and healthy individuals were native Japanese living in Japan. All patients with SLE fulfilled the American College of Rheumatology criteria for SLE [16]. This study was reviewed and approved by the research ethics committees of each participating institute, Sagamihara Hospital Research Ethics Committee, Nagasaki Medical Center Research Ethics Committee, Yokohama Minami Kyosai Hospital Research Ethics Committee, Tama Medical Center Research Ethics Committee, University of Tsukuba Research Ethics Committee, Kitasato University Research Ethics Committee, Komagome Hospital Research Ethics Committee, Teikyo University Research Ethics Committee, Himeji Medical Center Research Ethics Committee, Morioka Hospital Research Ethics Committee, Kyushu Medical Center Research Ethics Committee, Nagoya Medical Center Research Ethics Committee, Yokohama City University Medical Center Research Ethics Committee, the University of Tokyo Research Ethics Committee and Juntendo University Research Ethics Committee. Written informed consent was obtained from all study participants. This study was conducted in accordance with the principles expressed in the Declaration of Helsinki.

### Genotyping

Genotyping of *HLA-DRB1* and *DQB1* was performed by a polymerase chain reaction technique using sequence-specific oligonucleotide probes (WAKFlow HLA typing kits, Wakunaga, Hiroshima, Japan), using a Bio-Plex 200 system (Bio-Rad, Hercules, CA), or using MPH-2 HLA typing kits (Wakunaga). Results of *HLA-DRB1* genotyping for some of the healthy controls were reported previously [17], [18]. Although a small part of second set SLE patients recruited at University of Tsukuba or Juntendo University was overlapped with that in another study which reported susceptible effects of DRB1*09:01 [14], DNA collection and *HLA-DRB1* genotyping were independently performed for this replication study of protective association with DRB1*13:02 and *14:03. *DRB1-DQB1* haplotypes were elucidated by direct counting.

### Statistical analysis

Differences of allele carrier frequencies, genotype frequencies, haplotype carrier frequencies or amino acid residue carrier frequencies were analyzed by Fisher’s exact test using 2×2 contingency tables. Adjustment for multiple comparisons was performed using the Bonferroni method. Corrected *P* (*P*c) values were calculated by multiplying the *P* value by the number of alleles, haplotypes or amino acid residues tested. Relative predispositional effects (RPE) were analyzed by sequential elimination of carriers of each allele with the strongest association [19]. Correction for multiple testing of SLE with different clinical and phenotypic manifestations was performed by calculating false discovery rate Q-value [20].

## Results

### Association analysis of the first set of SLE patients and healthy controls

*HLA-DRB1* and *DQB1* genotyping was performed in 459 SLE patients and 307 healthy controls in the first set to compare carrier frequencies of each allele or haplotype (Table 1, left column, Table S1). Although the positive association between the carrier frequency of DRB1*15:01 and SLE failed to reach significance in this set (*P*c = 0.0705, odds ratio [OR] 1.76, 95% confidence interval [CI] 1.22–2.53, Table 1), a significant protective association was found for DRB1*13:02 and SLE (*P*c = 0.0123, OR 0.42, 95% CI 0.26–0.68). Additionally, the carrier frequency of the DRB1*14:03 allele was lower in SLE, although this difference was not statistically significant (*P*c = 0.0812, OR 0.30, 95% CI 0.14–0.68). The DR6 (DRB1*13 and *14) allele carrier frequency was significantly lower in SLE (*P* = 4.34×10^−5^, OR 0.51, 95% CI 0.37–0.70), but DR2 (DRB1*15 and *16) was not different (*P* = 0.1743, OR 1.23). A significant negative association was found for DQB1*06:04 and SLE (*P*c = 0.0499, OR 0.46, 95% CI 0.28–0.77, Table S1). The DRB1*13:02-DQB1*06:04 haplotype was negatively associated with SLE (*P*c = 0.0414, OR 0.30, 95% CI 0.26–0.72). Thus, we detected certain *DRB1 and DQB1* alleles which either conferred protection against or susceptibility to SLE.

**Table 1:** *HLA-DRB1* allele carrier frequency in the SLE patients and controls.

	1^st^ Set						2^nd^ set						combined						
	Case (n = 459)	Control (n = 307)	*P*	OR	*P*c	95%CI	Case (n = 389)	Control (n = 542)	*P*	OR	*P*c	95%CI	Case (n = 848)	Control (n = 849)	*P*	OR	*P*c	95%CI	*P* (RPE)
DRB1*01:01	41 (8.9)	27 (8.8)	1.0000	1.02	NS		34 (8.7)	65 (12.0)	0.1312	0.70	NS		75 (8.8)	92 (10.8)	0.1921	0.80	NS		
DRB1*04:01	19 (4.1)	4 (1.3)	0.0292	3.27	0.8189	(1.10–9.71)	15 (3.9)	15 (2.8)	0.3546	1.41	NS		34 (4.0)	19 (2.2)	0.0371	1.82	NS	(1.03–3.23)	
DRB1*04:03	15 (3.3)	15 (4.9)	0.2612	0.66	NS		16 (4.1)	29 (5.4)	0.4403	0.76	NS		31 (3.7)	44 (5.2)	0.1560	0.69	NS		0.0117
DRB1*04:05	94 (20.5)	68 (22.1)	0.5890	0.91	NS		79 (20.3)	125 (23.1)	0.3357	0.85	NS		173 (20.4)	193 (22.7)	0.2621	0.87	NS		
DRB1*04:06	17 (3.7)	26 (8.5)	0.0062	0.42	0.1738	(0.22–0.78)	10 (2.6)	36 (6.6)	0.0053	0.37	0.1533	(0.18–0.76)	27 (3.2)	62 (7.3)	0.0002	0.42	0.0052	(0.26–0.66)	0.0020
DRB1*04:10	15 (3.3)	10 (3.3)	1.0000	1.00	NS		10 (2.6)	15 (2.8)	1.0000	0.93	NS		25 (2.9)	25 (2.9)	1.0000	1.00	NS		
DRB1*08:02	59 (12.9)	31 (10.1)	0.2549	1.31	NS		40 (10.3)	32 (5.9)	0.0176	1.83	0.5114	(1.13–2.96)	99 (11.7)	63 (7.4)	0.0029	1.65	0.0848	(1.18–2.30)	0.0082
DRB1*08:03	93 (20.3)	50 (16.3)	0.1855	1.31	NS		61 (15.7)	76 (14.0)	0.5119	1.14	NS		154 (18.2)	126 (14.8)	0.0673	1.27	NS		0.0151
DRB1*09:01	139 (30.3)	72 (23.5)	0.0394	1.42	NS	(1.02–1.97)	118 (30.3)	151 (27.9)	0.4208	1.13	NS		257 (30.3)	223 (26.3)	0.0670	1.22	NS		
DRB1*11:01	11 (2.4)	18 (5.9)	0.0192	0.39	0.5378	(0.18–0.85)	15 (3.9)	16 (3.0)	0.4639	1.32	NS		26 (3.1)	34 (4.0)	0.3576	0.76	NS		
DRB1*12:01	37 (8.1)	20 (6.5)	0.4836	1.26	NS		36 (9.3)	43 (7.9)	0.4771	1.18	NS		73 (8.6)	63 (7.4)	0.3730	1.18	NS		
DRB1*12:02	12 (2.6)	6 (2.0)	0.6330	1.35	NS		14 (3.6)	24 (4.4)	0.6156	0.81	NS		26 (3.1)	30 (3.5)	0.6839	0.86	NS		
DRB1*13:02	30 (6.5)	44 (14.3)	0.0004	0.42	0.0123	(0.26–0.68)	39 (10.0)	93 (17.2)	0.0022	0.54	0.0646	(0.36–0.80)	69 (8.1)	137 (16.1)	5.21×10^−7^	0.46	1.51×10^−5^	(0.34–0.63)	7.17×10^−5^
DRB1*14:03	9 (2.0)	19 (6.2)	0.0029	0.30	0.0812	(0.14–0.68)	5 (1.3)	21 (3.9)	0.0247	0.32	0.7156	(0.12–0.86)	14 (1.7)	40 (4.7)	0.0004	0.34	0.0127	(0.18–0.63)	0.0010
DRB1*14:05	14 (3.1)	11 (3.6)	0.6836	0.85	NS		10 (2.6)	28 (5.2)	0.0635	0.48	NS		24 (2.8)	39 (4.6)	0.0715	0.60	NS		
DRB1*14:06	9 (2.0)	12 (3.9)	0.1176	0.49	NS		4 (1.0)	11 (2.0)	0.2962	0.50	NS		13 (1.5)	23 (2.7)	0.1284	0.56	NS		
DRB1*14:54	33 (7.2)	20 (6.5)	0.7727	1.11	NS		18 (4.6)	29 (5.4)	0.6521	0.86	NS		51 (6.0)	49 (5.8)	0.8375	1.04	NS		
DRB1*15:01	119 (25.9)	51 (16.6)	0.0025	1.76	0.0705	(1.22–2.53)	98 (25.2)	65 (12.0)	3.07×10^−7^	2.47	8.90×10^−6^	(1.75–3.49)	217 (25.6)	116 (13.7)	5.48×10^−10^	2.17	1.59×10^−8^	(1.69–2.79)	5.48×10^−10^
DRB1*15:02	69 (15.0)	63 (20.5)	0.0514	0.69	NS		76 (19.5)	116 (21.4)	0.5118	0.89	NS		145 (17.1)	179 (21.1)	0.0414	0.77	NS	(0.61–0.98)	
DR2 (DRB1*15, *16)	187 (40.7)	110 (35.8)	0.1743	1.23			170 (43.7)	183 (33.8)	0.0026	1.52		(1.16–1.99)	357 (42.1)	293 (34.5)	0.0014	1.38		(1.13–1.68)	
DR6 (DRB1*13, *14)	98 (21.4)	107 (34.9)	4.34×10^−5^	0.51		(0.37–0.70)	83 (21.3)	180 (33.2)	6.68×10^−5^	0.55		(0.40–0.74)	181 (21.3)	287 (33.8)	1.06×10^−8^	0.53		(0.43–0.66)	

SLE: systemic lupus erythematosus, OR: odds ratio, CI: confidence interval, *P*c: corrected *P* value, NS: not significant, RPE: relative predispositional effects. Allele carrier frequencies are shown in parenthesis (%). Alleles with more than 1% of the frequency in controls are shown. Association was tested by Fisher's exact test using 2×2 contingency tables. RPE were tested by sequential elimination of carriers of each of the alleles DRB1*15:01, *13:02, *14:03, *04:06, *08:02, *08:03 and *04:03.

### Replication in the second set and combined analysis

We then sought to confirm these findings in an independent set of SLE patients and healthy controls by *DRB1* genotyping the second set of 389 SLE patients and 542 healthy controls (Table 1, central column). In this case, a significant positive association was found for DRB1*15:01 and SLE (*P*c = 8.90×10^−6^, OR 2.47, 95% CI 1.75–3.49) and it was the protective association with DRB1*13:02 which just failed to achieve significance (*P*c = 0.0646, OR 0.54, 95% CI 0.36–0.80). As in the first set, the protective association with DRB1*14:03 also failed to reach significance (*P*c = 0.7156, OR 0.32, 95% CI 0.12–0.86). Carrier frequencies of DR6 (*P* = 6.68×10^−5^, OR 0.55, 95% CI 0.40–0.74) and DR2 (*P* = 0.0026, OR 1.52, 95% CI 1.16–1.99) alleles were significantly lower and higher, respectively, in SLE.

We further explored associations between these *DRB1* alleles and SLE in a combined analysis, using RPE testing [19]. RPE were analyzed by sequential elimination of carriers of each allele with the strongest association (Table 1, right column). The strongest association was between DRB1*15:01 and SLE, confirmed in this combined analysis (*P* = 5.48×10^−10^, *P*c = 1.59×10^−8^, OR 2.17, 95% CI 1.69–2.79). The second round of comparisons was conducted after the elimination of DRB1*15:01 carriers, revealing the next strongest association to be between DRB1*13:02 and SLE (*P* = 7.17×10^−5^, *P*c = 0.0020). The third round was after the elimination of both DRB1*15:01 or *13:02 carriers, now showing the strongest association to be between DRB1*14:03 and SLE (*P* = 0.0010, *P*c = 0.0272). Further rounds after elimination of DRB1*15:01, *13:02 or *14:03 carriers revealed only tendential associations between the remaining *DRB1* alleles and SLE, particularly for DRB1*04:06 (*P* = 0.0020, *P*c = 0.0509), *08:02 (*P* = 0.0082, *P*c = 0.2040), *08:03 (*P* = 0.0151, *P*c = 0.3615) and *04:03 (*P* = 0.0117, *P*c = 0.2683). We therefore focused on the *DRB1* alleles with the strongest SLE associations, namely DRB1*15:01, *13:02 and *14:03.

### Genotype analysis of HLA-DRB1*15:01, *13:02 and *14:03

We next compared genotype frequencies of HLA-DRB1*15:01, *13:02 and *14:03 to seek associations with SLE (Table 2). A significant positive association was found for “DRB1*15:01/allele other than *15:01” (*P* = 4.83×10^−9^, OR 2.14, 95% CI 1.65–2.76). On the other hand, “DRB1*13:02/allele other than *13:02” and the “*13:02/*13:02” genotypes were both negatively associated with SLE (*P* = 6.47×10^−6^, OR 0.49, 95% CI 0.36–0.67 and *P* = 0.0211, OR 0.11, 95% CI 0.01–0.87, respectively). A negative association of the “*13:02 or *14:03/alleles other than *13:02 or *14:03” genotype with SLE was also observed (*P* = 5.53×10^−9^, OR 0.43, 95% CI 0.33–0.58), again although a significant positive association for the genotype “alleles other than *13:02 or *14:03/alleles other than *13:02 or *14:03” was present (*P* = 3.69×10^−10^, OR 2.41, 95% CI 1.82–3.19). A significant positive association was found with “*15:01/alleles other than *13:02 or *14:03” (*P* = 1.79×10^−11^, OR 2.39, 95% CI 1.84–3.10). Thus, protective effects of *13:02 and *14:03 are dominant over the predisposing effects of *15:01 in SLE.

**Table 2:** *HLA-DRB1* genotype frequency in the SLE patients and controls.

	Case (n = 848)	Control (n = 849)	*P*	OR	95%CI
*15:01/alleles other than *15:01	198 (23.3)	106 (12.5)	4.83×10^−9^	2.14	(1.65–2.76)
*15:01/*15:01	19 (2.2)	10 (1.2)	0.0958	1.92	
*13:02/alleles other than *13:02	68 (8.0)	128 (15.1)	6.47×10^−6^	0.49	(0.36–0.67)
*13:02/*13:02	1 (0.1)	9 (1.1)	0.0211	0.11	(0.01–0.87)
*15:01/*13:02	8 (0.9)	12 (1.4)	0.5009	0.66	
*15:01/alleles other than *15:01 or *13:02	190 (22.4)	94 (11.1)	3.30×10^−10^	2.32	(1.77–3.03)
*14:03/alleles other than *14:03	13 (1.5)	40 (4.7)	0.0002	0.31	(0.17–0.59)
*14:03/*14:03	1 (0.1)	0 (0.0)	0.4997	3.01	
*15:01/*14:03	2 (0.2)	3 (0.4)	1.0000	0.67	
*13:02/*14:03	0 (0.0)	1 (0.1)	1.0000	0.33	
*15:01/alleles other than *15:01, *13:02 or *14:03	188 (22.2)	91 (10.7)	1.58×10^−10^	2.37	(1.81–3.11)
*13:02 or *14:03/any alleles	83 (9.8)	176 (20.7)	3.69×10^−10^	0.41	(0.31–0.55)
*13:02 or *14:03/alleles other than *13:02 or *14:03	81 (9.6)	166 (19.6)	5.53×10^−9^	0.43	(0.33–0.58)
*13:02 or *14:03/*13:02 or *14:03	2 (0.2)	10 (1.2)	0.0379	0.20	(0.04–0.91)
alleles other than *13:02 or *14:03/alleles other than *13:02 or *14:03	765 (90.2)	673 (79.3)	3.69×10^−10^	2.41	(1.82–3.19)
*15:01/*13:02 or *14:03	10 (1.2)	15 (1.8)	0.4209	0.66	
*15:01/alleles other than *13:02 or *14:03	207 (24.4)	101 (11.9)	1.79×10^−11^	2.39	(1.84–3.10)

SLE: systemic lupus erythematosus, OR: odds ratio, CI: confidence interval. Genotype frequencies are shown in parenthesis (%). Association was tested by Fisher's exact test using 2×2 contingency tables.

### Associations of DRB1 with SLE of different clinical and phenotypic manifestations

We analyzed the associations of genotype frequencies of *15:01, *13:02 and *14:03 separately in SLE patients with different clinical and autoantibody phenotypes to confirm the protective effects of *13:02 and *14:03 in SLE patients with different manifestations. The significant positive association of the genotype “*15:01/alleles other than *13:02 or *14:03” with SLE of a certain phenotype was confirmed for almost all factors assessed (Table 3, left column). The same was true for the negative association of the “*15:01/*13:02 or *14:03” genotype with SLE regardless of phenotype (Table 3, right column). Thus, the protective effects of *13:02 and *14:03 were confirmed in all the different phenotypic manifestations of SLE.

**Table 3:** *HLA-DRB1* genotype frequency in the SLE patients and controls relative to SLE phenotype.

	n	*15:01/alleles other than *13:02, or *14:03	*P*	OR	*Q*	95%CI	*15:01/*13:02, or *14:03	*P*	OR	*Q*	95%CI
age of onset < 20	144	36 (25.0)	7.58×10^−5^	2.47	0.0001	(1.60–3.80)	1 (0.7)	0.4920	0.39	0.8806	
anti-Ro/SS-A antibodies (+)	326	80 (24.5)	2.13×10^−7^	2.41	6.04×10^−7^	(1.74–3.34)	4 (1.2)	0.6138	0.69	0.8806	
anti-La/SS-B antibodies (+)	70	14 (20.0)	0.0591	1.85	0.0628		1 (1.4)	1.0000	0.81	1.0000	
anti-RNP antibodies (+)	240	60 (25.0)	1.62×10^−6^	2.47	3.07×10^−6^	(1.72–3.53)	1 (0.4)	0.2192	0.23	0.8806	
anti-Sm antibodies (+)	220	51 (23.2)	5.14×10^−5^	2.23	7.49×10^−5^	(1.53–3.25)	2 (0.9)	0.5478	0.51	0.8806	
anti-dsDNA antibodies (+)	603	148 (24.5)	5.53×10^−10^	2.41	3.13×10^−9^	(1.82–3.18)	7 (1.2)	0.3916	0.65	0.8806	
antiphospholipid syndrome (+)	165	37 (22.4)	0.0007	2.14	0.0009	(1.41–3.26)	1 (0.6)	0.4925	0.34	0.8806	
malar rash (+)	301	90 (25.4)	2.08×10^−8^	2.52	8.85×10^−8^	(1.83–3.45)	2 (0.7)	0.2655	0.37	0.8806	
discoid rash (+)	116	84 (27.9)	5.28×10^−10^	2.87	3.13×10^−9^	(2.07–3.97)	2 (1.7)	1.0000	0.98	1.0000	
photosensitivity (+)	238	28 (24.1)	0.0007	2.36	0.0009	(1.47–3.78)	3 (1.3)	0.7770	0.71	0.8806	
arthritis (+)	410	60 (25.2)	1.44×10^−6^	2.50	3.06×10^−6^	(1.74–3.57)	4 (1.0)	0.3336	0.55	0.8806	
serositis (+)	161	96 (23.4)	3.46×10^−7^	2.26	8.41×10^−7^	(1.66–3.08)	0 (0.0)	0.1477	0.17	0.8806	
renal disorder (+)	380	35 (21.7)	0.0015	2.06	0.0017	(1.34–3.16)	5 (1.3)	0.6354	0.74	0.8806	
neurologic disorder (+)	130	93 (24.5)	7.01×10^−8^	2.40	2.38×10^−7^	(1.76–3.28)	3 (2.3)	0.7219	1.31	0.8806	
hemolytic anemia (+)	95	35 (26.9)	1.84×10^−5^	2.73	3.14×10^−5^	(1.76–4.24)	2 (2.1)	0.6857	1.20	0.8806	
lymphopenia (+)	459	18 (18.9)	0.0708	1.73	0.0708		4 (0.9)	0.2335	0.49	0.8806	
thrombocytopenia (+)	180	122 (26.6)	5.58×10^−11^	2.68	9.49×10^−10^	(2.00–3.60)	2 (1.1)	0.7513	0.62	0.8806	
Control	849	101 (11.9)					15 (1.8)				

SLE: systemic lupus erythematosus, OR: odds ratio, CI: confidence interval. Genotype frequencies are shown in parenthesis (%). Associations were tested by Fisher's exact test using 2×2 contingency tables. To correct for multiple testing, the false discovery rate Q-value was calculated.

### Amino acid residues in the DRβ chain are associated with SLE

**Figure 1:**
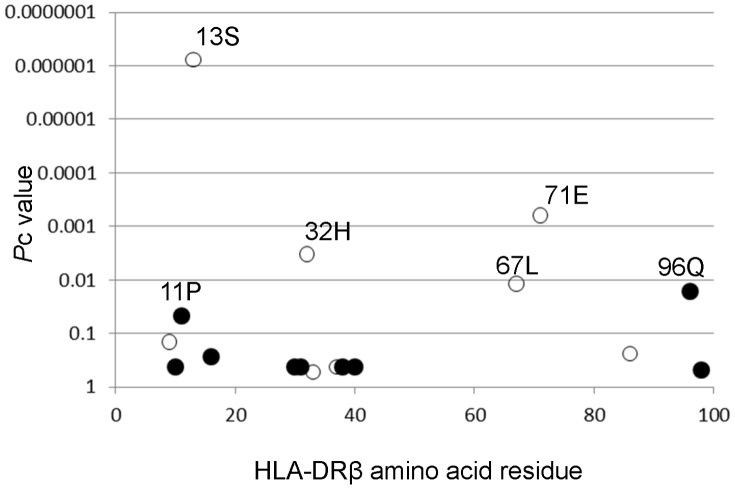
Associations of amino acid residues in the DRβ chain with SLE. Corrected *P* (*P*c) values were calculated by multiplying the *P* value by the number of amino acid residues tested. Associations were established by Fisher’s exact test using 2×2 contingency tables. Positive associations were indicated in filled circles and negative in open circles.

Amino acid residues in the HLA-DRβ chain were also analyzed for associations with SLE. The amino acid residue 13S in the DRβ chain showed strong protective associations with SLE (*P* = 2.24×10^−8^, *P*c = 7.63×10^−7^, OR 0.55, 95%CI 0.45–0.68, Figure 1) and is shared by *13:02 and *14:03, whereas the amino acid residues 32H (*P* =  9.76×10^−5^, *P*c = 0.0033, OR 0.67, 95%CI 0.55–0.82), 67L (*P* =  0.0003, *P*c = 0.0118, OR 0.70, 95%CI 0.58–0.85), and 71E (*P* = 1.84×10^−5^, *P*c = 0.0006, OR 0.53, 95%CI 0.40–0.71) showed only moderate protective associations and are possessed by *13:02 and *14:03, *14:03, and *13:02, respectively. Thus, association analysis suggested roles for certain defined amino acid residues in the DRβ chain in SLE susceptibility.

## Discussion

Several studies have shown that certain *HLA-DR* alleles are positively associated with SLE. However, few studies have focused on negative associations of *HLA* alleles with SLE. To the best of our knowledge, this is the first report of a negative association of the HLA-DR6 alleles DRB1*13:02 and *14:03 with Japanese SLE, although a lower frequency of DR5 [21] or DR6 [22] alleles in Asian patients with SLE has been reported before. Several studies have noted positive associations of DRB1*15:01 [11], [21], [23], *15:02 [12], [13] or *09:01 [3], [14], [24], [25] alleles with SLE in Asians. However, here we only confirmed the association with *15:01, but not *15:02 or *09:01 in Japanese SLE patients in general (Table 1). The association of *15:02 with SLE has never been reported in a Japanese population, suggesting that this allele may not be the primary genetic factor in itself, but a marker for a nearby gene. Since the allele frequencies between Thai and Japanese are comparable [13], [18], the differences in allele frequencies could not explain the reason of the lack of association of *15:02 in Japanese SLE. Because HLA is the strongest genetic factor for SLE, it is quite difficult to explain the reason by other genetic backgrounds than *HLA* region. Since the amino acid sequences of *15:01 and *15:02 are almost same, presented peptide will be same. Environmental background could not explain the reason. HLA region is in strong linkage disequilibrium. Therefore, we cannot rule out the possibility that another causative genes, namely *DRB5* or *DQA1* genes, might exist in the *HLA* region in linkage disequilibrium with the culprit gene in the *DRB1* locus.

In the genotype analysis of HLA-DRB1*15:01, *13:02 and *14:03, a nominally lower frequency of the “*15:01/*13:02 or *14:03” genotype in SLE was observed, although a positive association was revealed for the genotype of “*15:01/alleles other than *13:02 or *14:03” (Table 2). The protective effects of *13:02 or *14:03 thus seem to overcome the predispositional effects of *15:01 in SLE. DRB1*13:02 commonly belongs to the haplotype DRB1*13:02-DQB1*06:04-DPB1*04:01 positively selected in Japanese in recent history [26]. The DRB1*13:02 allele is also a protective allele for cervical cancer caused by human papilloma virus infection [27] and DPB1*04:01 is protective for hepatitis B infection [28].

The *DRB1* and *DQB1* alleles which showed significant associations are in strong linkage disequilibrium. In order to elucidate which of the *DRB1* and *DQB1* genes was responsible for primary association, haplotype analysis of *DRB1-DQB1* was performed. A significant negative association was found for the DQB1*06:04 allele and the DRB1*13:02-DQB1*06:04 haplotype with SLE in the first set comparison (Table S1). The primary role of *DRB1* or *DQB1* was not elucidated, because the strong linkage disequilibrium between DRB1*13:02 and DQB1*06:04 results in a low frequency of DRB1*13:02 in patients without DQB1*06:04 and similarly a low frequency of DQB1*06:04 in patients without DRB1*13:02.

It was reported that anti-Ro/SS-A-positive rheumatoid arthritis patients were more frequently DRB1*08:03-positive and an association of DRB1*15:01 and anti-La/SS-B antibodies has been reported in Japanese rheumatoid arthritis patients [29]. In the present study, a significant positive association of DRB1*04:05 with the presence of anti-Ro/SS-A antibodies in SLE patients was found; in contrast, the DRB1*12:02 allele was associated with the presence of anti-La/SS-B antibodies. These four *DRB1* alleles are in linkage disequilibrium with DPB1*05:01 in the Japanese population [30], suggesting a role for *DPB1*in the production of autoantibodies to ribonucleoprotein. Susceptibility alleles for background diseases, DRB1*15:01 in SLE or DRB1*04:05 in rheumatoid arthritis, might not be easily detected as autoantibody-associated alleles in a comparison of antibody-positive and -negative patients. Alternatively, these findings could be explained by differences in the pathogenesis of rheumatoid arthritis and SLE.

Amino acid residues 13, 32, 67 and 71 of the HLA-DRβ chain were found to be associated with SLE (Figure 1). Residues 13, 32, 67 and 71 form the HLA-DR peptide-binding groove [31]. These data suggest the involvement of peptide antigens bound to specific HLA molecules in controlling the development of SLE.

The negative association with *HLA-DR6* alleles needs to be confirmed in future independent studies. Because the allelic distribution of *HLA* in other ethnic populations is different from the Japanese, the protective role of some *DRB1* alleles in SLE in other populations should be determined.

This is the first identification of a negative association of HLA-DRB1*13:02 and *14:03 with SLE. Our findings support the dominantly protective role of *HLA-DR6* alleles in the pathogenesis of SLE.

## Supporting Information

Table S1 ***HLA-DQB1*** **allele and** ***DRB1-DQB1*** **haplotype carrier frequency in the 1st set of SLE patients and controls.**
(PDF)
