# Supplementary material for: Human Leukocyte Antigens and Systemic Lupus Erythematosus: A Protective Role for the HLA-DR6 Alleles DRB1*13:02 and *14:03
Source: PLoS One. 2014 Feb 3;9(2):e87792. doi: 10.1371/journal.pone.0087792 (PMC3912000; doi:10.1371/journal.pone.0087792)
Supplement: Table S1 — HLA-DQB1 allele and DRB1-DQB1 haplotype carrier frequency in the 1st set of SLE patients and controls. (PDF) [file pone.0087792.s001.pdf]

Supplementary Table 1 *HLA-DQB1* allele and *DRB1-DQB1* haplotype carrier frequency in the 1st set of SLE patients and controls.

DQB1 allele	Case (n=459)	Control (n=307)	*P*	OR	*P*c	95%CI
*DQB1*03:01*	87 (19.0)	73 (23.8)	0.1230	0.75	NS	
*DQB1*03:02*	64 (13.9)	66 (21.5)	0.0079	0.59	0.1102	(0.40-0.86)
*DQB1*03:03*	154 (33.6)	78 (25.4)	0.0163	1.48	0.2282	(1.07-2.05)
*DQB1*04:01*	92 (20.0)	67 (21.8)	0.5858	0.90	NS	
*DQB1*04:02*	41 (8.9)	23 (7.5)	0.5082	1.21	NS	
*DQB1*05:01*	47 (10.2)	27 (8.8)	0.5350	1.18	NS	
*DQB1*05:02*	28 (6.1)	11 (3.6)	0.1335	1.75	NS	
*DQB1*05:03*	28 (6.1)	25 (8.1)	0.3098	0.73	NS	
*DQB1*06:01*	153 (33.3)	107 (34.9)	0.6972	0.93	NS	
*DQB1*06:02*	114 (24.8)	49 (16.0)	0.0039	1.74	0.0540	(1.20-2.52)
*DQB1*06:04*	28 (6.1)	38 (12.4)	0.0036	0.46	0.0499	(0.28-0.77)
DRB1-DQB1 haplotype						
**01:01-*05:01*	41 (8.9)	27 (8.8)	1.0000	1.02	NS	
**04:01-*03:01*	18 (3.9)	4 (1.3)	0.0449	3.09	NS	(1.04-9.23)
**04:03-*03:02*	14 (3.1)	15 (4.9)	0.2460	0.61	NS	
**04:05-*03:02*	1 (0.2)	4 (1.3)	0.1635	0.17	NS	
**04:05-*04:01*	91 (19.8)	65 (21.2)	0.6485	0.92	NS	
**04:06-*03:02*	17 (3.7)	25 (8.1)	0.0095	0.43	0.3431	(0.23-0.82)
**04:07-*03:02*	4 (0.9)	3 (1.0)	1.0000	0.89	NS	
**04:10-*04:02*	15 (3.3)	7 (2.3)	0.5114	1.45	NS	
**07:01-*02:01*	3 (0.7)	3 (1.0)	0.6884	0.67	NS	
**08:02-*04:02*	27 (5.9)	14 (4.6)	0.5132	1.31	NS	
**08:02-*03:02*	28 (6.1)	17 (5.5)	0.8756	1.11	NS	
**08:03-*03:01*	3 (0.7)	2 (0.7)	1.0000	1.00	NS	
**08:03-*06:01*	88 (19.2)	48 (15.6)	0.2469	1.28	NS	
**09:01-*03:01*	2 (0.4)	3 (1.0)	0.3949	0.44	NS	
**09:01-*03:02*	1 (0.2)	1 (0.3)	1.0000	0.67	NS	
**09:01-*03:03*	137 (29.8)	69 (22.5)	0.0250	1.47	0.8988	(1.05-2.05)
**11:01-*03:01*	9 (2.0)	16 (5.2)	0.0206	0.36	0.7417	(0.16-0.83)
**11:01-*03:02*	0 (0.0)	2 (0.7)	0.1603	0.13	NS	
**12:01-*03:01*	26 (5.7)	11 (3.6)	0.2295	1.62	NS	
**12:01-*03:03*	11 (2.4)	6 (2.0)	0.8050	1.23	NS	
**12:02-*03:01*	11 (2.4)	6 (2.0)	0.8050	1.23	NS	
**13:01-*06:03*	8 (1.7)	4 (1.3)	0.7710	1.34	NS	
**13:02-*06:04*	28 (6.1)	40 (13.0)	0.0012	0.43	0.0414	(0.26-0.72)
**13:02-*06:09*	1 (0.2)	4 (1.3)	0.1635	0.17	NS	
**14:03-*03:01*	9 (2.0)	19 (6.2)	0.0029	0.30	0.1044	(0.14-0.68)
**14:05-*05:03*	14 (3.1)	11 (3.6)	0.6836	0.85	NS	
**14:06-*03:01*	9 (2.0)	12 (3.9)	0.1176	0.49	NS	
**14:54-*05:03*	13 (2.8)	13 (4.2)	0.3136	0.66	NS	
**14:54-*05:02*	20 (4.4)	8 (2.6)	0.2418	1.70	NS	
**15:01-*03:01*	4 (0.9)	1 (0.3)	0.6533	2.69	NS	
**15:01-*06:02*	115 (25.1)	50 (16.3)	0.0041	1.72	0.1462	(1.19-2.49)
**15:02-*06:01*	66 (14.4)	63 (20.5)	0.0301	0.65	NS	(0.44-0.95)
**16:02-*05:02*	8 (1.7)	3 (1.0)	0.5397	1.80	NS	

SLE: systemic lupus erythematosus, OR: odds ratio, *P*c: corrected *P* value, CI: confidence interval. Allele carrier or haplotype carrier frequencies are shown in parentheses (%). Alleles with more than 1% of the frequency, or haplotypes with more than 0.1% of the frequency in controls are shown. Association was tested by Fisher's exact test using 2X2 contingency tables.
